# Integrated Transcriptomic and Proteomic Analysis Reveals Up-Regulation of Apoptosis and Small Heat Shock Proteins in Lens of Rats Under Low Temperature

**DOI:** 10.3389/fphys.2021.683056

**Published:** 2021-06-17

**Authors:** Jiayue Zhou, Jing Wu, Sifan Zheng, Xiangjun Chen, Daizhan Zhou, Xingchao Shentu

**Affiliations:** ^1^The Eye Center, Second Affiliated Hospital of School of Medicine, Zhejiang University, Hangzhou, China; ^2^Zhejiang Provincial Key Lab of Ophthalmology, Second Affiliated Hospital of School of Medicine, Zhejiang University, Hangzhou, China; ^3^GKT School of Medical Education, King’s College London, London, United Kingdom; ^4^Institute of Translational Medicine, Zhejiang University School of Medicine, Hangzhou, China

**Keywords:** hypothermia, lens, transcriptomics, proteomics, small heat shock proteins, apoptosis

## Abstract

Cold cataract is the reversible opacification of the lens when the temperature decreases. However, we observed that when temperature of the rats’ lens was maintained at a lower temperature for a prolonged time, the opacification of lens was only partly reversible. To review the potential molecular mechanism of the irreversible part of opacification under cold stimulation, we applied comparative transcriptomic and proteomic analysis to systematically investigate the molecular changes that occurred in the lens capsules of rats under low temperature treatments. The RNA sequencing based transcriptomic analysis showed a significant up-regulation of genes related to the lens structure and development in the Hypothermia Group. Hub genes were small heat shock proteins (sHSPs). Besides the same findings as the transcriptomic results, the liquid chromatography-tandem mass spectrometry based proteomic analysis also revealed the up-regulation of the apoptotic process. To further analyze the regulatory mechanism in this process, we subsequently performed integrated analysis and identified the down-regulation of Notch3/Hes1 and PI3K/Akt/Xiap signaling axis. Our research revealed the activation of the apoptotic process in rats’ lens under cold stimulation, and the sHSP related heat shock response as a potential protective factor through our transcriptomic and proteomic data.

## Introduction

Cold cataract is a phenomenon described as a reversible opacification of the lens when the temperature decreases (Zigman and Lerman, 1964), and has been observed in the lenses of young rats, mice, fishes, cows, and humans (Zigman and Lerman, 1964; Benedek et al., 1979; Loewenstein and Bettelheim, 1979). Early studies revealed that the reversible part of the opacification was mainly caused by the temporary aggregation of γ crystalline (Zigman and Lerman, 1964; Siezen et al., 1985) or liquid-liquid phase separation (Tanaka et al., 1977). The severity of cold cataract was positively correlated with time and negatively correlated with temperature (Bermudez et al., 2011). However, we observed that when temperature of the rats’ lens was maintained at a lower temperature (4°C) for a prolonged time (8 h), the transparency of lens did not fully recover, and the tension of lens capsules increased. There lacks mechanistic research regarding the formation of these irreversible opacifications caused by hypothermal stimulation.

The lens is one of the most important refractive mediums in the eye. Keeping the lens transparent is important for vision and for accurate observation of the inside of the eye. The lens has no blood vessels or nerves, and the lens fiber cells in the core of the lens have degraded their organelles, including the nuclei (Bassnett et al., 2011). Lens epithelial cells (LECs) located under the anterior lens capsule are the most metabolically active part of the lens. LECs act as a barrier between the aqueous humor and send signals to the inside of the lens. LECs play a major role in maintaining lenses’ life activities and transparency. Abnormal changes and apoptosis of LECs are often observed across different types of cataract.

To identify the potential molecular mechanism of irreversible opacifications under cold treatment, we used RNA-seq and TMT based LC-MS/MS to systematically investigate the transcriptomic and proteomic changes in lens capsules of rats under low temperature treatments. To further analyze the regulatory mechanism in this process, we subsequently performed integrated analysis of the transcriptomic and proteomic changes.

## Materials and Methods

### Animal and Tissue Collection

All animal experiments complied with the Association for Research in Vision and Ophthalmology Statement for the Use of Animals in Ophthalmic and Vision Research. All animals were treated in accordance with the Zhejiang University Administration on Laboratory Animal Care. Male Sprague-Dawley rats (6 weeks, 200 g) were obtained from Shanghai SLAC Laboratory Animal Co., Ltd.

**FIGURE 1:**
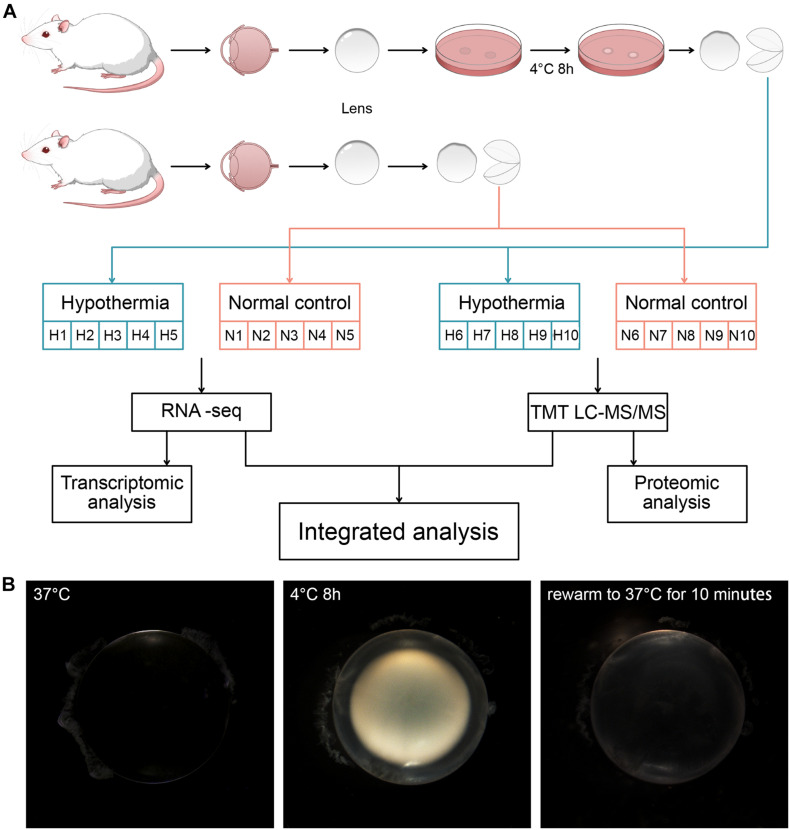
**(A)** Grouping of the rats and treatments of their lens. Strategy for transcriptomic and proteomic analysis of the lens capsules in each group. **(B)** The morphological changes induced by hypothermia and rewarming treatments.

Rats were divided randomly into the Normal Group and the Hypothermia Group and subsequently euthanized. The lens capsules of rats in the Normal Group were collected directly after euthanasia. The lens of rats in Hypothermia Group were isolated and incubated in Hibernate-A medium (Thermo Fisher Scientific, MA, United States) at 4°C for 8 h. The lens capsules were separated from the lens cortex after being washed in 4°C phosphate-buffered solution (PBS). For RNA-seq and TMT, 5 rats were used from each group. Two lens capsules from a rat were combined as a sample (Figure 1A). The collected lens capsules were rapidly frozen in liquid nitrogen and stored at −80°C for further transcriptomic and proteomic analysis. For RT-qPCR, 6 rats were used from each group. 4 lens capsules from 2 rats were combined as a sample. The samples were put into a TRIzol reagent (Invitrogen) immediately after separation and stored at 4°C.

### RNA Sequencing

#### Sample Preparation

Total RNA of the Hypothermia Group (*n* = 5) and the Nomal Control Group (*n* = 5) were extracted using TRIzol reagent (Invitrogen, Carlsbad, CA, United States). Agarose gel electrophoresis, NanoPhotometer spectrophotometer (IMPLEN, CA, United States) and RNA Nano 6000 Assay Kit of the Bioanalyzer 2100 system (Agilent Technologies, CA, United States) were used for total RNA qualification. RNA samples with RIN ≥ 5.8 were select for sequencing.

#### Library Preparation

A total amount of 0.8 μg RNA per sample was used as input material for the library preparation. Sequencing libraries were generated using NEBNext^®^ Ultra^TM^ RNA Library Prep Kit for Illumina^®^ (NEB, United States) following manufacturer’s recommendations.

#### Sequencing

The library preparations were sequenced using the Novaseq 6000 platform (Illumina, United States) and 150 bp paired-end reads were generated.

#### Data Analysis

Clean data were obtained by removing reads containing adapter, reads containing ploy-N and low-quality reads from the raw data of fastq format. Q20, Q30 and GC content of the clean data were calculated at the same time. Paired-end clean reads were aligned to the rat reference genome Rnor_6.0 using Hisat2 (v2.0.5), featurecounts (v1.5.0-p3) was used to count the number of reads mapped to each gene. And then FPKM (expected number of Fragments Per Kilobase of transcript sequence per Millions base pairs sequenced) of each gene was calculated based on the length of the gene and reads count mapped to this gene. Differential expression analysis of two groups was performed using DESeq2 v1.16.1. *P*-values were adjusted using Benjamini and Hochberg’s approach. Genes with | log2FoldChange | > 1 compared with Normal Control group and adjusted *P* < 0.05 were considered as DEGs for transcriptomic analysis.

### Liquid Chromatography-Tandem Mass Spectrometry (LC-MS/MS) Proteomic

Sample preparation: Samples were lysed and digested using pressure cycling technology (PCT), as described previously with modifications (Zhu and Guo, 2018). The lysis buffer contained 6 M urea (Sigma) and 2 M thiourea (Sigma) in 100 mM triethylammonium bicarbonate (TEAB, Thermo Fisher Scientific). The peptide concentration was measured using a ScanDrop spectrophotometer (Endress + Hauser Group, Lorrach, Germany).

Tandem Mass Tag (TMT) Labeling and Off-Line High pH Reversed-Phase Fractionation: Peptides were labeled with TMT 10-plex label reagents (Thermo Fisher Scientific, San Jose, United States) as described previously (Gao et al., 2020; Shen et al., 2020). Peptides were separated into 120 fractions, which were consolidated into 30 fractions. The fractions were subsequently dried and redissolved in 2% ACN/0.1% formic acid (FA).

DDA-MS Acquisition: The redissolved peptides were analyzed by LC-MS/MS with the Ultimate 3000 nanoLC-MS/MS system (Dionex LC-Packings, CA) coupled with a Q Exactive HF-X mass spectrometer (QE-HFX, Thermo Fisher Scientific) in data dependent acquisition (DDA) mode as described previously (Shen et al., 2020).

Data Analysis: The resultant MS data were analyzed with Proteome Discoverer (Version 2.4.1.15, Thermo Fisher Scientific) using a protein database composed of the Rat fasta database downloaded from UniProtKB on 12 May 2020, containing 9949 reviewed protein sequences. Enzyme, static modifications, precursor ion mass toleranceas were set as described previously (Shen et al., 2020). The peptide-spectrum-match allowed 1% target false discovery rate (FDR) (strict) and 5% target FDR (relaxed). Normalization was performed against the total peptide amount. Default setup was used for all other parameters. We used two tailed two-sided *t*-tests to identify the differential expression proteins between two groups. Differential expression proteins were defined by the thresholds based on adjusted *P* < 0.05, | log2FoldChange | > 0.25.

### Integrated Analysis of Transcriptomic and Proteomic Results

Overlapped genes in the lists of DEGs and DEPs were taken to perform correlation analysis according to their log2FoldChanges using Prism v8.2.1 and PPI analysis using String v11.0. List of rat translation factors (TFs) and cofactors (co-TFs) was download from AnimalTFDB3.0 to identify the TFs or co-TFs in the overlapped genes. Then, KEGG analysis of these TFs and co-TFs was performed.

### Real-Time Quantitative PCR

Total RNAs of the lens capsule samples of Hypothermia Group (*n* = 3) and the Nomal Control Group (*n* = 3) were extracted using Trizol Reagent (Thermo Fisher), four corneas served as one biological replicate. RNA concentrations and OD260/OD280 ratios (1.9–2.0) were determined using a Nanodrop 1,000 UV spectrophotometer (Thermo Fisher Scientific, Waltham, MA, United States). Approximately 1,500 ng RNA was used per reverse transcription reaction (PrimeScriptRT Reagent Kit; TaKaRa, Shiga, Japan). The TB Green Premix Ex Taq Kit (TaKaRa) was used for quantitative real-time PCR on a 7500 Fast Real-Time PCR System (ABI, Loma Linda, CA, United States), with a 2-step method. The expression levels of the target genes were normalized to that of β-actin and were calculated using the 2^–ΔΔCT^ method and indicated as means ± SE, statistical analysis was conducted using *t*-test. The data are presented as fold-changes. The primers (TSINGKE, Beijing, China) are shown in Supplementary Table 1.

### Data Analysis and Visualization

Gene Ontology (GO) and KEGG pathway enrichment analysis of differentially expressed genes (DEGs) implemented by The Database for Annotation, Visualization and Integrated Discovery (DAVID v6.8). The cut off value was FDR < 0.01 and results were visualized by R software (v3.6.1). Protein-protein interaction (PPI) analysis was implemented by String (v11.0), and visualized by Cytoscape (v3.7.2). The node degree were calculated using cytoHubba plugin in Cytoscape(v3.7.2)., and the gene with node degree ≥ 10 was set as the hub gene in PPI network.

## Results

### Irreversible Opacification in the Lens of Sprague-Dawley Rats After 8 h of Cold Treatment

The newly extracted lens of rats at normal body temperature (37°C) was completely transparent. After 8 h of low temperature treatment at 4°C, the lens showed severe opacification. Following rewarming to 37°C for 10 min, the cold cataract disappeared. However, compared with the lens before cold treatment, the whole lens still had irreversible opacity (Figure 1B).

### Transcriptomic Analysis Reveals Up-Regulation of Lens-Specific Gene Expression and Small Heat Shock Proteins (sHSPs) in Cold Treated Lens Capsules

In order to identify the differential expressed mRNA under cold treatment, we compared the transcriptomic changes of rats’ lens capsules between the Hypothermia Group and the Normal Group. Approximately 45 million paired-end clean reads per sample on average were obtained, and mapped to a total number of 17,005 expressed genes.

**FIGURE 2:**
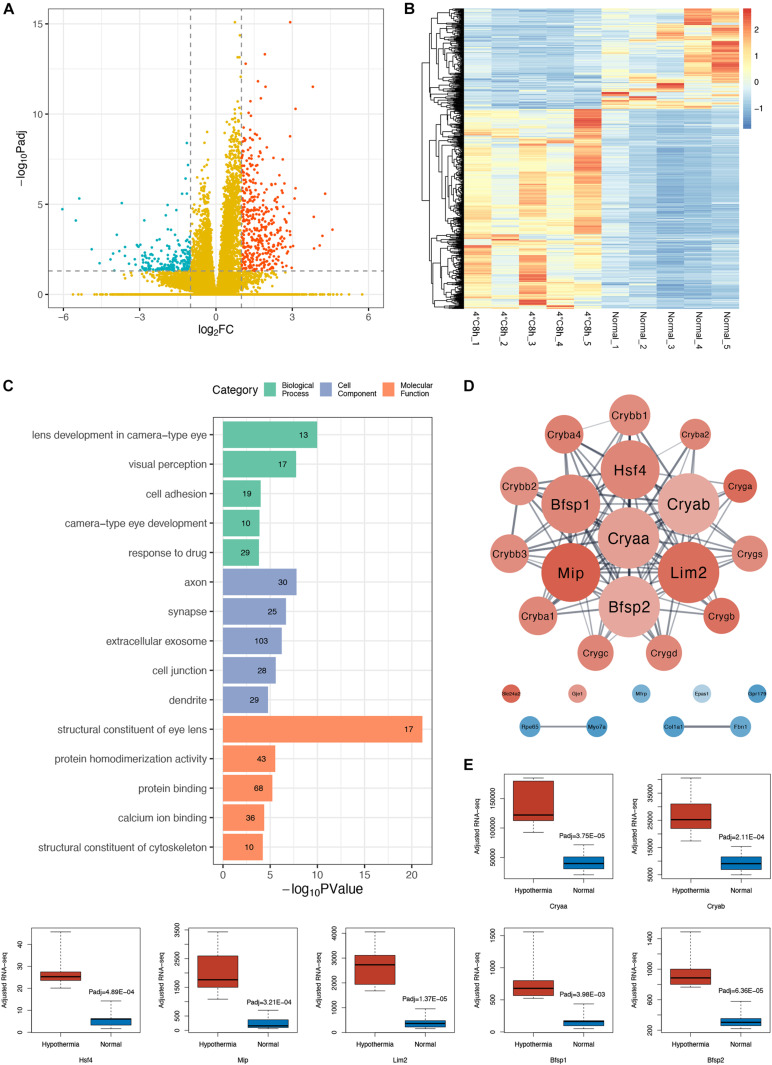
Analysis of transcriptomic changes in cold treated lens capsules. **(A)** Volcano plots depict the log2FoldChange and statistical significance (|log2FoldChange| > 1), with the adjusted *P*-value expressed as −log10(adjusted *P*-value), the *P*-values were adjusted using the Benjamini and Hochberg method. Red points, green points, and yellow points represent up-regulated genes, down-regulated genes, and non-differentially expressed genes, respectively. **(B)** Heatmap of differentially expressed genes (DEGs) in the 10 samples. **(C)** Gene ontology (GO) analysis bar plot of DEGs. The top five GO terms with the lowest FDR values within each subcategory (BP, CC, MF) are listed. The number of DEGs annotated to the GO terms are shown on the bars. **(D)** Protein-protein interaction (PPI) network of DEGs annotated to the GO terms related to eyes. **(E)** Comparison of gene expression level of the hub genes in the PPI network between the two groups, adjusted RNA-seq means FPKM.

DEGs analysis identified 627 significant changed genes between the two groups (adjusted *P* < 0.05, | log_2_FoldChange | > 1), of which 420 genes were up-regulated and 207 genes were down-regulated (Figures 2A,B).

To find out the main functions of the DEGs and the biological processes they are involved in, we performed a GO enrichment analysis. The GO enrichment results suggested that many significantly enriched terms of the DEGs were related to the eye and the lens, such as lens development in camera type eye (GO:0002088, FDR = 2.13E-07), visual perception (GO:0007601, FDR = 1.91E-05), structural constituent of eye lens (GO:0005212, FDR = 1.48E-18) (Figure 2C). We subsequently performed a PPI analysis of DEGs enriched in those GO terms related to the eye (Figure 2D). We found that the gene cluster consisted of genes specifically expressed in the lens, including α, β, γ crystallin families, membrane protein Mip and Lim2, cytoskeleton protein Bfsp1, and Bfsp2. All these genes were up-regulated (Figure 2E). Surprisingly, among the hub genes, Cryaa and Cryab are sHSPs and Hsf4 is a heat shock transcriptome factor.

GO enrichment analysis of up-regulated and down-regulated DEGs separately shows interesting results. Up-regulated DEGs were enriched in the eye-related and cytoskeleton-related categories (Supplementary Table 2). Down-regulated DEGs were enriched in more comprehensive categories including cell adhesion, extracellular matrix, ion transmembrane transport, and response to stimuli such as drugs, starvation, wounds, and TGF beta (Supplementary Table 3).

### Proteomic Changes Correspond With Transcriptomic Changes and Show the Up-Regulation of the Apoptotic Process

**FIGURE 3:**
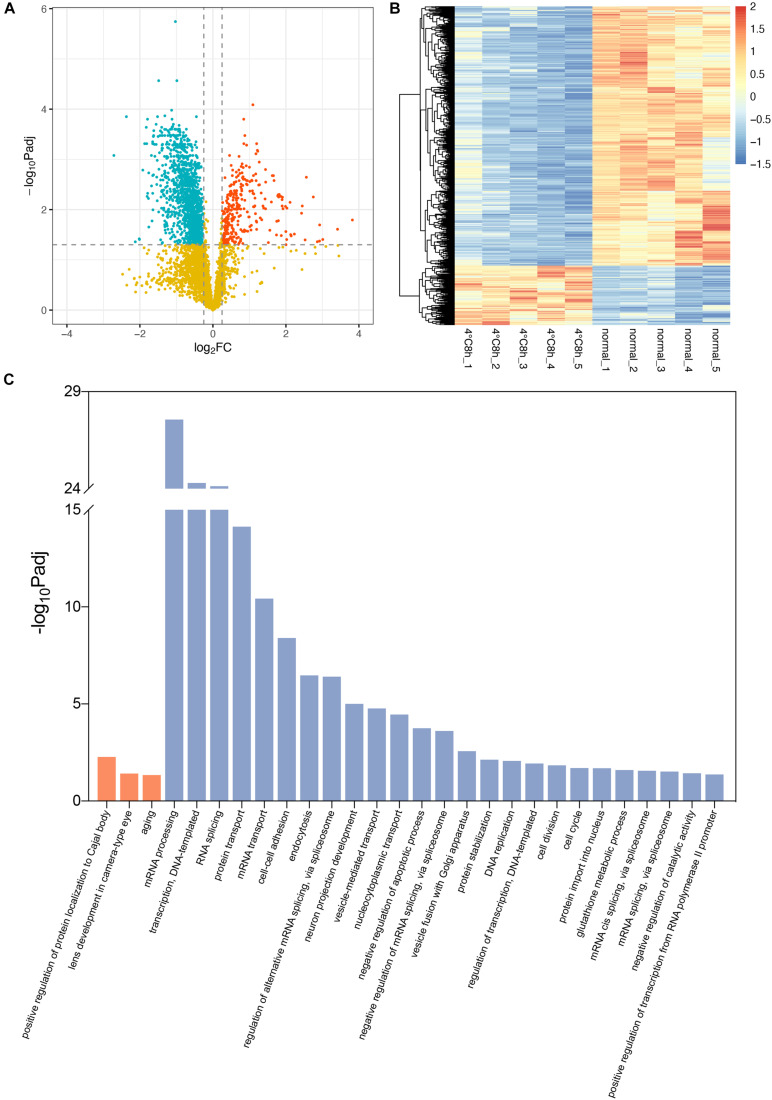
Analysis of proteomic changes in cold treated lens capsules. **(A)** Volcano plots depict the log2FoldChange and statistical significance, with the adjusted *P*-value expressed as −log10(adjusted *P*-value). Red points, green points, and yellow points represent up-regulated genes, down-regulated genes, and non-differentially expressed genes, respectively. **(B)** Heatmap of differentially expressed proteins (DEPs) in the 10 samples. **(C)** Biological process in Gene ontology (GO) analysis for the up- and down-regulated DEPs. Terms in orange bars were enriched from up-regulated proteins and terms in blue bars were enriched from down-regulated proteins.

A total of 4,149 proteins were identified in the 10 samples. A total of 1,641 of these proteins were significant differentially expressed proteins (DEPs) (adjusted *P* < 0.05, | log2FoldChange | > 0.25). Among these DEPs, 306 were up-regulated and 1,335 were down-regulated (Figures 3A,B).

Using the cut-off value of the adjusted *P*-value (Bonferroni) < 0.05, up-regulated DEPs were enriched in 3 significant biological processes Gene Ontology (GO) terms, while down-regulated DEPs were enriched in 25 terms. The results suggested the up-regulated DEPs were enriched in “lens development in camera-type eye” (GO:0002088, Padj = 0.039) in the category of “Biological Process.” The associated DEPs are Cryaa, Cryab, Cryba1, Crygb, Crygc, Crygs, and Mip, which are the same as the results of RNA-seq. “Structural constituent of eye lens” (GO:0005212, Padj = 7.21E-16) in the category of “Molecular Function” contains even more lens specific DEPs including Bfsp1 and Bfsp2 (Supplementary Table 3). Conversely, GO terms “negative regulation of apoptotic process” (GO:0043066, Padj = 1.77E-04), “cell division” (GO:0051301, Padj = 0.014), “cell cycle” (GO:0007049, Padj = 0.020) in which down-regulated DEPs are enriched, indicates the up-regulation of apoptosis and the stagnation of cell cycle and division (Figure 3C).

### Integrated Analysis of Transcriptomic and Proteomic Results Reveals the Up-Regulation of Apoptosis

**FIGURE 4:**
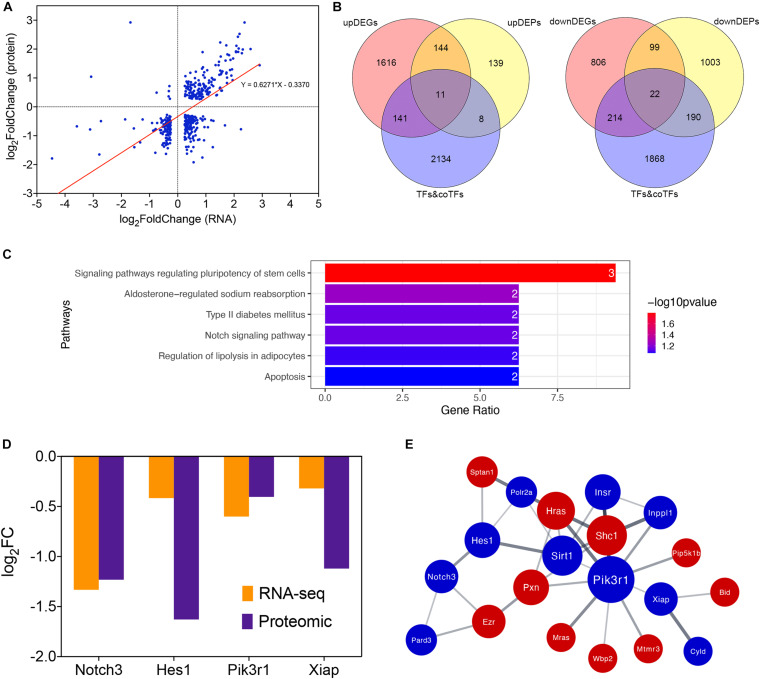
Integrated analysis of transcriptomic and proteomic changes in cold treated lens capsules of the Hypothermia Group. **(A)** Correlationship between transcriptomic and proteomic results, the horizontal coordinate indicates the log2FoldChange of DEGs, while the vertical coordinate indicatesthe log2FoldChange of DEPs. **(B)** Overlap of differentially expressed genes (DEGs, differentially expressed proteins (DEPs, and translation factors (TFs) and cofactors (co-TFs) database, up-regulated and down-regulated part were separated. **(C)** KEGG pathway enrichment analysis of differentially expressed TFs&co-TFs. **(D)** RNA and protein expression level of the four TFs&co-TFs in the Notch signaling pathway and Apoptosis KEGG pathways. **(E)** PPI network of Notch3, Hes1, Pik3r1, and Xiap and their first neighbors in the PPI network of DEGs and DEPs regulated in the same direction.

Protein expression levels are usually determined by the corresponding mRNA expression levels. To verify this regulatory relationship, we performed an integrated analysis of our transcriptomic and proteomic results. There were 421 overlapped genes belonging to both DEGs and DEPs in our study (adjusted *P* < 0.05, | log_2_FoldChange | > 0.25). Correlation analysis shows that the expression changing of protein and mRNA were positively correlated in general (*r* = 0.627, *p* < 0.0001; Figure 4A). Therefore, transcriptional regulation plays a major role when the lens is stimulated by hypothermia. We also compared the genes regulated in same direction in RNA and proteins with a database of translation factors (TFs) and cofactors (co-TFs) (Hu et al., 2019). 33 TFs or co-TFs were identified, among which 11 were up-regulated, and 22 were down-regulated (Figure 4B). To identify the key TFs and pathways, we performed a KEGG pathway enrichment analysis of these 33 genes The Notch signaling pathway (rno04330, including gene Notch3 and Hes1) and the Apoptosis pathway (rno04210, including gene Pik3r1 and Xiap) were identified (Figure 4C). We found that Notch3, Hes1, Pik3r1 and Xiap were all down-regulated DEGs and DEPs (Figure 4D). PPI of these four genes and their first neighbors in the PPI network of DEGs and DEPs regulated in the same direction shows a tight interaction (Figure 4E).

### Confirmation of Gene Expression by RT-qPCR

**FIGURE 5:**
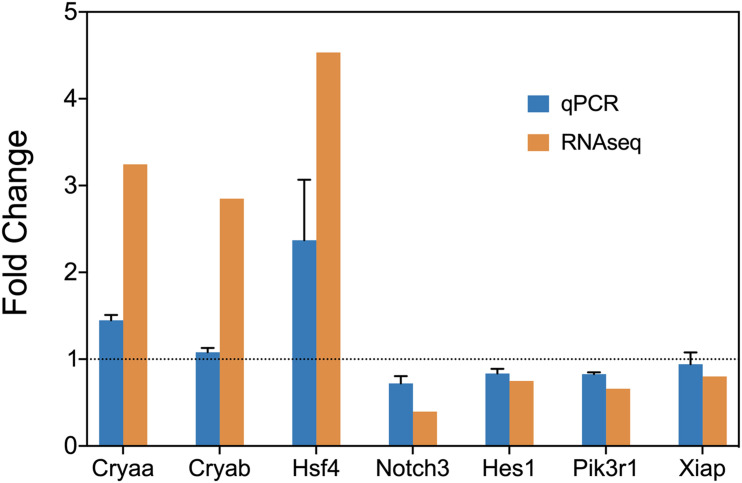
RT-qPCR confirmation of the expression level of Cryaa, Cryab, Hsf4, Notch3, Hes1, Pik3r1, and Xiap.

We used RT-qPCR to confirm the results above. sHSPs Cryaa, Cryab, heat shock transcription factor Hsf4, transcription factor Hes1, transcription co-factors Notch3, Pik3r1, Xiap were selected. The RT-qPCR confirmed the expression change of these genes (Figure 5).

## Discussion

We studied the transcriptomic and proteomic changes in cold-treated lens capsules of rats (Hypothermia Group) compared with the normal body temperature group (Normal Group. The transcriptomic and proteomic results showed high consistency as genes related to lens structure and development (α, β, γ crystallins, cytoskeletal protein BFSP1, and BFSP2, membrane proteins such as Mip and Lim2, and heat shock transcription factor Hsf4) were all significantly up-regulated. Based on the positive correlation between the transcriptomic and proteomic results, we hypothesized that transcriptional regulation played an important role and identified two apoptotic-related signaling pathways centered on four key regulatory factors. RT-qPCR was used to confirmed the gene expression level.

Previous studies have reported that mutations of these up-regulated genes in the lens were related to the formation of cataracts (Shiels and Hejtmancik, 2017). In addition to the membrane proteins Mip and Lim2 causing cataract formation by affecting cell permeability and adhesion (Louis et al., 1989; Ponnam et al., 2008; Kumari et al., 2013; Shentu et al., 2015), Cryaa, Cryab and Hsf4 are surprisingly all involved in the heat shock response. The α-crystallin subunits Cryaa and Cryab, also known as Hspb4 and Hspb5, are major protein components of the eye lens, and also belong to the sHSP (also called heat shock protein family B) family (Horwitz, 2003; Clark et al., 2012). sHSPs are a family of ATP-independent chaperones that can delay the onset of irreversible protein aggregation in response to cellular stressors (Janowska et al., 2019; Rosenzweig et al., 2019). α-crystallin appeared to have therapeutic effects in certain diseases. Injection of α-crystallin B prior to the intraocular pressure elevation markedly mitigated the neurodegenerative processes in rat glaucoma models (Anders et al., 2017). α-crystallin also protected axons from optic nerve degeneration in rats (Ying et al., 2008). In addition, we further screened our data and found that another two sHSPs, Hspb1 and Hspb2, were significantly up-regulated. Hspb1 could inhibit reactive oxygen species (ROS) and has a protection effect against atherosclerosis and coronary heart disease (Zhang et al., 2019). Hspb2 suppresses caspases-8 and 10 activation, thereby blocking downstream apoptotic events and inhibits apoptosis (Oshita et al., 2010). Upstream of these sHSPs is the heat shock transcription factor Hsf4, which is an important transcription factor in lens development and lens cell differentiation. Its target genes include the subunit of α, β, and γ crystallin, Bfsp1, Bfsp2 as well (Fujimoto et al., 2004; Shi et al., 2009). Hsf4 could protected human lens epithelial cells from oxidant stress by increasing the HMOX-1 expression in human lymphatic endothelial cells, which would then up-regulate antioxidant enzyme activity, and thus inhibit caspase family dependent apoptosis (Liao et al., 2018). In response to stress, the heat shock transcription factors can up regulate the cellular concentrations of some sHSPs (de Thonel et al., 2012; Zhong et al., 2016). sHSP Hspb1 and Cryab are target genes of Hsf4 (Fujimoto et al., 2004), which could be activated in response to cellular damage. Therefore, we postulate that the energy-saving heat shock response of sHSPs plays a major protective role in the homeostasis regulation of cold induced irreversible cataract formation.

In the integrated analysis of transcriptomic and proteomic results, we aimed to identify regulatory factors in the lens under cold stimulation, considering the positive correlation between the changing expression level of protein and mRNA. We discovered two important regulatory pathways, the anti-apoptotic pathway centered on Pik3r1 and Xiap, and the Notch signaling pathway centered on Notch3 and Hes1. Pik3r1 encodes the 85 kD regulatory subunit of Phosphoinositide-3-kinase (PI3K), which inhibits cell apoptosis through PI3K/Akt signaling pathway by activating NF-κB and inhibiting FOXO1 and BAD (Vivanco and Sawyers, 2002). The activation of PI3K/Akt signaling pathway was proven to inhibit oxidative stress and epithelial cell apoptosis in the lens of ultraviolet radiation-induced cataract rats (Yao and Yan, 2020). X-chromosome-linked inhibitor of apoptosis protein (Xiap) is the downstream target of Akt, which could be activated when Akt was phosphorylated (Castells et al., 2013). Xiap able to bind to and inhibit the caspases that mediate apoptotic cell death (Jost and Vucic, 2020). Akt1 and Akt2 were both detected as down-regulated in our proteomic data. Notch3 is a transmembrane, developmental signaling receptor, playing many crucial roles in developmental patterning, cell fate decisions, regulation of cell survival and proliferation (Hosseini-Alghaderi and Baron, 2020). Hes1 is one of the most studied targets of the Notch signaling pathway (Rani et al., 2016). Loss of Hes1 sensitizes mammalian cells to ER stress. Severe ER stress triggers cell death through expression of various pro-apoptotic genes (Lee et al., 2018). In a study of asthma pathogenesis, inhibition of Notch/Hes1 promotes PTEN expression (Li et al., 2020), which is an antagonist of PI3K/Akt pathway. On the contrary, promotion of the Notch3 production activated the downstream PI3K/Akt pathway and inhibited cardiomyocyte apoptosis, which alleviated cardiac ischemia/reperfusion injury (Zhang et al., 2015). Our results are consistent with those of past research, cold-induced apoptosis was observed in the myocardium (Kong et al., 2020) and rat hepatocytes (Vairetti et al., 2001). Apoptosis of lens epithelial cells was known to be an early event in the development of many types of cataracts (Li and Spector, 1996; Shiels and Hejtmancik, 2019). Previous studies suggested that the dominant-negative mutation of Pik3r1 could lead to cataract in human and mice (Solheim et al., 2017), and the activation of PI3K/AKT signaling pathway by circRNA HIPK3 or melatonin could inhibit apoptosis of HLECs (Bai et al., 2013; Cui et al., 2020). Notch2 was proved to be inhibited in the formation of age-related cataract (Fan et al., 2017). Hes1 was detected down-regulated in the cell model of glucocorticoid cataract (Gupta et al., 2005). Thus, the cold-induced irreversible cataract may be the result of lens epithelial cells apoptosis caused by the down regulation of Notch3/Hes1 and PI3K/Akt/Xiap signaling axis. Our study was a further proof of the importance of these two pathways in the formation of cataract. The down-regulated regulatory molecules Pik3r1, Xiap, Notch3, and Hes1 we’ve found out from our dataset need further research for their potential value.

In conclusion, the down-regulation of key molecules in the anti-apoptotic pathway and the Notch signaling pathway promotes the apoptosis of lens epithelial cells and promotes the occurrence of opacity. On the other hand, lens epithelial cells respond to cold stimulation through heat shock response. The expression of heat shock transcription factor Hsf4 was up-regulated, thereby enhancing the expression of sHSP family members.

We hypothesize that the balance was broken under cold stimulation. The up-regulated heat shock response was not strong enough to inhibit apoptosis successfully in physiological state, causing the lens to form irreversible opacity. There were some limitations of our study. First, the Normal Control Group was not treated with hibernation medium, but considering the RNA degradation of *in vitro* tissues at body temperature, we chose to collect the sample directly after euthanasia. Second, for some of the antibodies were difficult to obtain, we only verified the results by RT-qPCR. If possible, we would collect and sequence cold-treated lens capsule samples at more time points and different temperature in the future to obtain and analyze a series of constant data. Further research on the potential protective effects of the sHSPs and the anti-apoptotic Notch3/Hes1 and PI3K/Akt/Xiap signaling pathways in lens epithelial cells should also be considered.

## Data Availability Statement

The transcriptomic data presented in the study are deposited in the GEO repository, accession number GSE173481. The proteomic data presented in the study are deposited in the iProX repository, accession number IPX0002974000.

## Ethics Statement

The animal study was reviewed and approved by the Zhejiang University Administration on Laboratory Animal Care.

## Author Contributions

XS designed the study and revised the manuscript. JZ and JW designed the study, collected the samples, and drafted the manuscript. JZ and DZ analyzed the data. DZ, SZ, and XC revised the manuscript. All authors read and approved the final manuscript.

## Conflict of Interest

The authors declare that the research was conducted in the absence of any commercial or financial relationships that could be construed as a potential conflict of interest.
