# Supplementary figures and images for: Integrated Transcriptomic and Proteomic Analysis Reveals Up-Regulation of Apoptosis and Small Heat Shock Proteins in Lens of Rats Under Low Temperature
Source: Front Physiol. 2021 Jun 17;12:683056. doi: 10.3389/fphys.2021.683056 (PMC8247577; doi:10.3389/fphys.2021.683056)

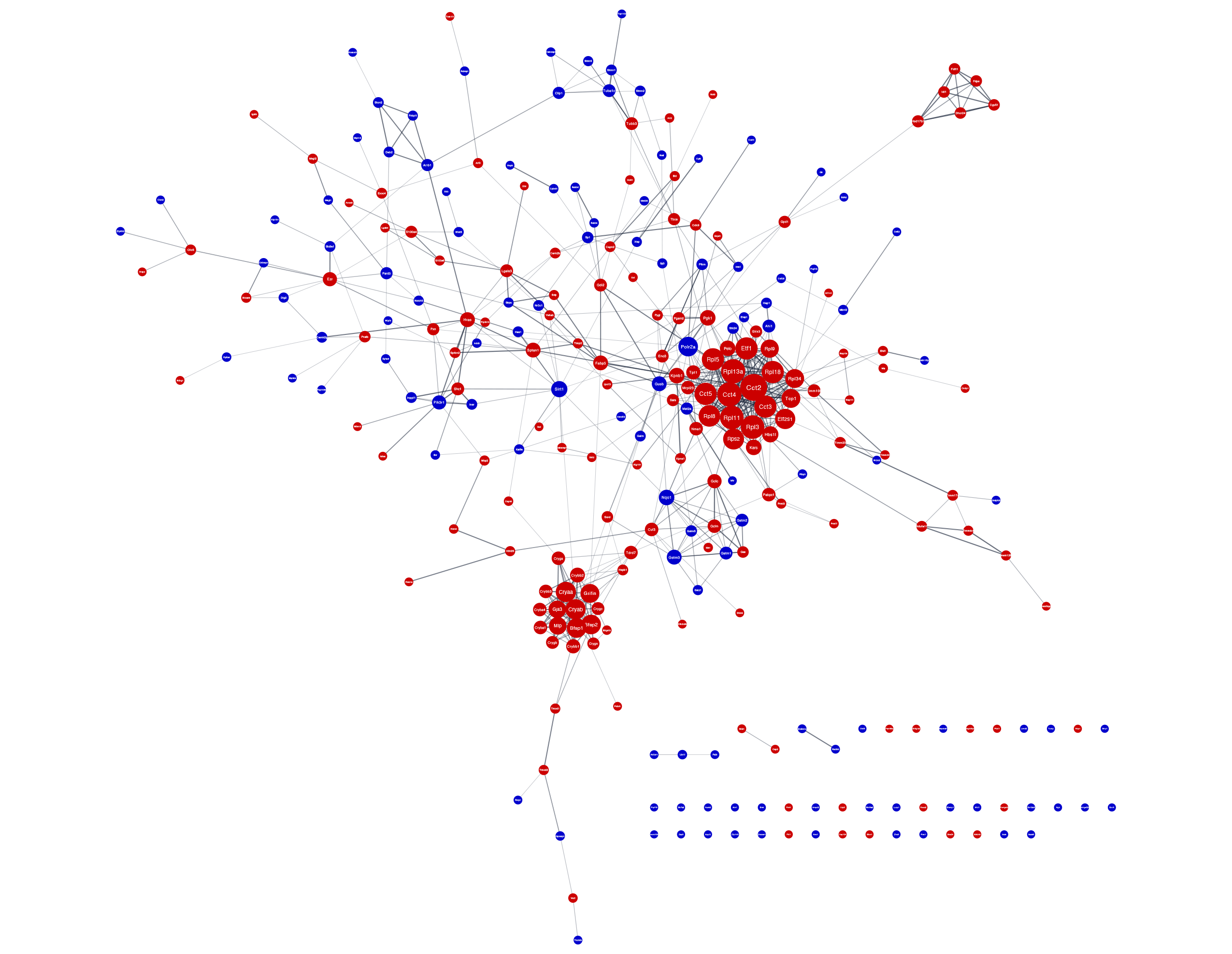

Supplement: Supplementary file 4 [file Image_1.TIF]
